# Shear Wave Elastography of the Plantar Fascia: Comparison between Patients with Plantar Fasciitis and Healthy Control Subjects

**DOI:** 10.3390/jcm10112351

**Published:** 2021-05-27

**Authors:** Daniel Baur, Christoph Schwabl, Christian Kremser, Mihra S. Taljanovic, Gerlig Widmann, Luca Maria Sconfienza, Judith Sztankay, Gudrun Feuchtner, Andrea S. Klauser

**Affiliations:** 1 Radiology Department, Medical University Innsbruck, Anichstrasse 35, 6020 Innsbruck, Austria; daniel.baur@i-med.ac.at (D.B.); christian.kremser@i-med.ac.at (C.K.); gerlig.widmann@tirol-kliniken.at (G.W.); gudrun.feuchtner@i-med.ac.at (G.F.); andrea.klauser@i-med.ac.at (A.S.K.); 2 Banner University Medical Center, Department of Medical Imaging, College of Medicine, The University of Arizona, Tucson, AZ 85724, USA; mihrat@radiology.arizona.edu; 3 Unit of Diagnostic and Interventional Radiology, I.R.C.C.S. Istituto Ortopedico Galeazzi, 20097 Milano, Italy; luca.sconfienza@unimi.it; 4 Dipartimento di Scienze Biomediche per la Salute, Università degli Studi di Milano, 20133 Milano, Italy; 5 Department of Radiotherapy, Medical University of Innsbruck, 6020 Innsbruck, Austria; judith.sztankay@tirol-kliniken.at

**Keywords:** color doppler ultrasonography, elastography, plantar fasciitis, plantar fascia, shear wave

## Abstract

Background: The purpose of this study was to evaluate plantar fasciae of healthy subjects and patients with plantar fasciitis by shear wave velocity (SWV) and stiffness with correlation to B-Mode and color Doppler ultrasonography (CDUS) and to establish cut-off values. Methods: This observational study was conducted with the approval of the Institutional Review Boards (IRBs) of our institution. 108 unilateral plantar fasciae were evaluated by including 87 consecutive patients (mean age 51.7; range: 29–82) (66 women and 21 men) with plantar fasciitis and 21 asymptomatic age matched healthy volunteers (mean age 47.3; range: 32–58) (15 women and 6 men). All patients were prospectively imaged between July 2018 and March 2019. B-mode US was used to measure thickness and CDUS to grade vascularity. SWE measurements were repeated 3 times and mean was used for statistical analysis. Results: Mean SWV value in healthy subjects was 6.94 m/s and in patients 4.98 m/s with a mean stiffness value of 152.88 kPa and 93.54 kPa respectively (*p* < 0.001). For SWV a cut-off value of 6.16 m/s had a specificity of 80.95% and sensitivity of 79.31%. For stiffness a cut-off value of 125.57 kPa had a specificity of 80.95% and sensitivity of 80.46%. No correlation to CDUS was found. The mean thickness of healthy fascias was 3.3 mm (range 2.4–3.9) compared to 6.1 mm (range 2.0–22.0) in plantar fasciitis (*p* < 0.001) with no correlation to SWV or to stiffness (r² = 0.02, *p* = 0.06). Conclusion: SWE allows quantitative assessment of plantar fascia stiffness, which decreases in patients with plantar fasciitis. No correlation to the thickness of the plantar fascia was found, therefore it represents an independent factor for the diagnosis of plantar fasciitis and could be helpful in addition to thickness measurement in unclear cases.

## 1. Introduction

Plantar fasciitis is the most common cause of heel pain in adults [1]. The lifetime prevalence may reach 10% of the general population worldwide. It has substantial im-pact on patients’ quality of life, which is shown by a study from Palomo-López et al., where the significance of health-related quality of life for foot pain, foot function, footwear, and general foot health, especially in women, was demonstrated [2,3]. Although the etiology of plantar fasciitis is multifactorial, mechanical overload and degeneration have been regarded as the main factors [4]. The plantar fascia seems to be affected even by foot deformities like hallus valgus deformity [5]. Therefore the importance of the plantar fascia as a main factor for foot health is evident. A prior systematic review revealed that increased age was also one of the factors associated with chronic plantar heel pain [6]. Age-related changes in tendons, as well as specific changes to the elastic modulus with degeneration, have been reported in prior studies [7,8,9] and may also occur in the plantar fascia [10].

Sonoelastography (SEL) is an ultrasound (US) imaging technique that allows for a noninvasive estimation of tissue stiffness [11]. It is based on the fact that softer tissue has greater tissue displacement than hard tissue when externally compressed. SEL allows calculation and comparison of tissue displacement before and after tissue compression with conventional US equipment but modified software and is used in MSK application [12,13].

Ultrasound-based shear wave elastography (SWE) allows for quantitative assessment of tissue stiffness [14,15]. Although it is well established in imaging of other regions, e.g., breast-imaging, the use in MSK imaging is still on the rise [16,17]. In the past 2 decades, sonoelastography has been progressively used as a tool to help evaluate soft-tissue elasticity and add information obtained with conventional gray-scale and Doppler ultrasonographic techniques. Recently introduced, SWE is considered to be more objective, quantitative, and reproducible than compression sonoelastography with increasing applications to the musculoskeletal system [13]. SWE uses an acoustic radiation force pulse sequence to generate shear waves, which propagate perpendicular to the ultrasound beam, causing transient displacements. SWE has a promising role in determining the severity of disease of various musculoskeletal tissues including tendons, muscles, nerves, and ligaments [12,13,14,15,18].

SEL has been applied to assess the stiffness of various tissues. It has been used to detect tendinopathy in the common extensor tendon of the elbow and the Achilles tendon [19,20,21]. Thus, SEL may add information about the mechanical properties of plantar fascia in addition to B-mode morphology.

SEL has been used previously in plantar fascia showing a softening in patients with plantar fasciitis [22,23]. SWE results for plantar fascia in healthy volunteers were reported by Chino et al. [24] and Wu et al. [22,25] however, to our knowledge there are only a few publication for SWE in plantar fasciitis, e.g., only preliminary results of Gatz et al. in 39 patients with plantar fasciitis [18]. The purpose of our study was to compare SWE of the plantar fascia between healthy subjects and patients with plantar fasciitis by shear wave velocity and stiffness with findings obtained by B-Mode and CDUS and to evaluate objective SWE parameters originating from the US system immanent calculation in a larger population, to gain new forms of information about the changes of the plantar fascia in plantar fasciitis, which could help in the diagnosis of unclear cases.

## 2. Materials and Methods

### 2.1. Ethical Consideration

All subjects gave their informed consent for inclusion before they participated in the study. The study was conducted in accordance with the Declaration of Helsinki, and the protocol was approved by the Institutional Review Boards (IRBs) of our institution (ethical approval code: 353/4.3).

### 2.2. Design and Sampling

The study is designed as an observational study with random sampling. The sampling size was sufficient. When considering 10% of the worldwide population we reached a CI of 95% with a margin of error of 6%. Healthy volunteers and patients were recruited from the trauma surgery department of the Medical University Innsbruck. Diagnosis of plantar fasciitis was based on the patient’s history and on results of the physical examination. Patients presented with inferior heel pain on weight bearing, pain persisting for ≥6 months with discomfort improving after further ambulation and worsening with continued activity, exacerbating pain when walking barefoot, on toes, or upstairs [26]. The participants had no treatment so far. Alternative diagnosis have been ruled out by trauma surgery specialists. If both feet were symptomatic, the more painful one was included in the evaluation. For healthy volunteers a random assessment of left or right heel was performed. The sonographer was blinded to clinical diagnosis and patients’ symptoms. US was used as the first-line imaging examination.

### 2.3. Study Population

108 unilateral plantar fasciae (81 women and 27 men) with normal physical activity were evaluated by including 87 consecutive patients (mean age 51.7, range: 29.0–82.0) (66 women and 21 men) with plantar fasciitis and 21 asymptomatic healthy volunteers recruited from our hospital staff (mean age 47.3, range: 32–58) (15 women and 6 men). All patients were prospectively imaged between July 2018 and March 2019.

**Inclusion criteria** comprised the presence of unilateral heel pain at the origin of the plantar fascia on the medial tubercle of the calcaneus lasting for more than 6 month.

**Exclusion criteria** included affected patients because of Morbus Ledderhosen (*n* = 1), Achilles tendinosis (*n* = 1), any treatment for plantar fasciitis (*n* = 1) or any previous surgery of the examined foot (*n* = 2), stress fracture or tumors.

### 2.4. Data Recollection

US examination: All US studies were performed by a single radiologist with three years of experience in SWE. The experiments were carried out in the Department of Rheumatology- and Sports Imaging, Medical University Hospital Innsbruck. Each US examination was performed using a SuperLinear™ SL12-7 MHz transducer with a bandwidth of 7–12 MHz (SuperSonic Imagine’s Aixplorer^®^, SuperSonic Imagine, 510 rue René Descartes, Les jardins de la Duranne Bât. F, 13857, Aix-en-Provence, France). Each examination was performed according to a standardized protocol with patients placed in a prone position, legs extended with their feet on a positioning role. Feet were kept relaxed during all measurements, hanging free over the examination bed in a 90 degree angle.

#### 2.4.1. B-Mode US Examination

**Figure 1:**
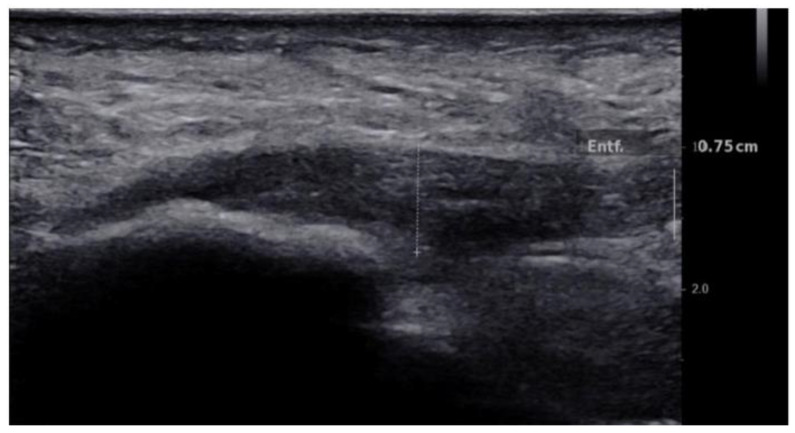
Longitudinal B-mode ultrasound image of a 50-year-old patient with plantar fascia shows hypoechogenicity and thickening of the plantar fascia of 7 mm.

Maximum thickness (in mm, craniocaudal dimension) of each plantar fascia was measured in the longitudinal plane (i.e., perpendicular to the direction of the fibers) at the insertion of the plantar fascia at the calcaneus. (Figure 1).

#### 2.4.2. CDUS Examination

Accompanied hypervascularity modified according to Fenwick et al. was graded as per a semi-quantitative grading system consisting of 4 grades: Grade 0 = no vascularity (=normal), Grade 1 = 1/3 hypervascularity in the fascia, Grade 2 = 2/3 hypervascularity in the fascia, Grade 3 = 3/3 hypervascularity in the fascia [27]. CDUS was performed with standardized machine settings by using a frequency of 7 MHz with a pulse repetition frequency of 750 to 1000 kHz, a low wall filter, and medium persistence. The window (colour box) was restricted to the plantar fascia. After visualization of colour-flow signals, pulsed wave spectral Doppler imaging was performed using the lowest filter setting and the smallest scale available that would display the Doppler waveforms as large as possible without aliasing. A spectral Doppler tracing was obtained to confirm that the CDUS signals represented true arterial or venous flow

#### 2.4.3. SWE Examination

**Figure 2:**
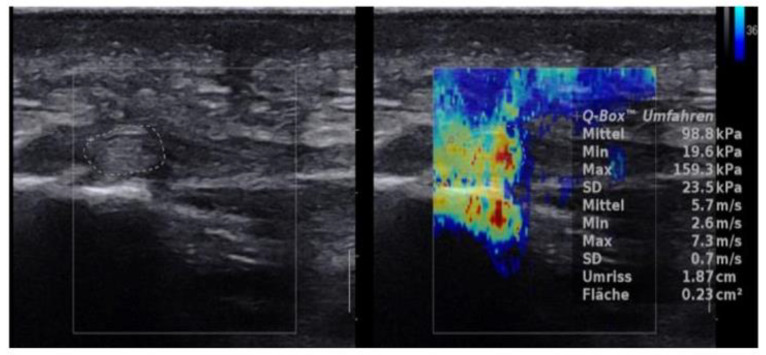
Longitudinal SWE image and B-mode ultrasound image of a 50-year-old patient with normal plantar fascia demonstrates stiffness of 98.8 kPa and SWV value of 5.7 m/s.

**Figure 3:**
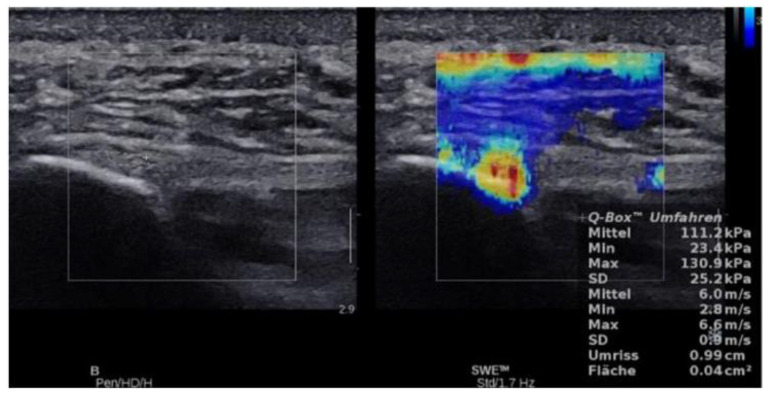
Longitudinal SWE image and B-mode ultrasound image of a 30 years old healthy volunteer with normal plantar fascia demonstrates stiffness of 111.2 kPa and SWV value of 6.0 m/s.

For stiffness and shear wave velocity three SWE measures were obtained at the same session in the longitudinal plane by manual tracking of a ROI which was repeated 3 times after unfreezing and freezing the SWE result. B-mode was used to longitudinally align the transducer with the plantar fascia. The transducer was kept stationary with light pressure on top of a generous amount of coupling gel for 4–5 s during the acquisition of each SWE sonogram. For each SWE sonogram, the ROI was tracked manually centered on the plantar fascia, ensuring that the diameter of the ROI was within the thickest part of the plantar fascia. For further analysis, the mean of the three measurements was used. The stiffness and SWE values were given in kPa and m/s, respectively, and tabulated. (Figure 2 and Figure 3)

### 2.5. Statistical Analysis

Statistical analysis was performed using R Project for Statistical Computing 3.4.1. Core Team, written by Robert Gentleman and Ross Ihaka of the Statistics Department of the University of Auckland. For the three repeated SWE measurements, intra-rater variability was determined by calculating the intra-class correlation coefficient using the irr package for R (Various Coefficients of Interrater Reliability and Agreement. R package version 0.84, by Matthias Gamer). The Shapiro-Wilk normality test was used to check for normal distribution. As patient data turned out to be normally distributed but not data for healthy subjects a Wilcoxon signed rank was used for group comparisons. To obtain cut-off values to distinguish between healthy and patient group receiver operating characteristic (ROC) analysis was applied using the pROC package for R (Robin X, Turck N, Hainard A, et al. (2011) pROC: an open-source package for R and S+ to analyze and compare ROC curves. BMC Bioinformatics (12:77). For analyzing correlation between SWE data and thickness values as determined by B-mode US a linear model was fitted to the data and the coefficient of determination (r²) calculated. A coefficient (r) of <0.3 showed no correlation, 0.3–0.5 a weak correlation, 0.5–0.7 a moderate correlation and 0.7–1 a high correlation. *p*-values of <0.05 were considered statistically significant.

## 3. Results

**Figure 4:**
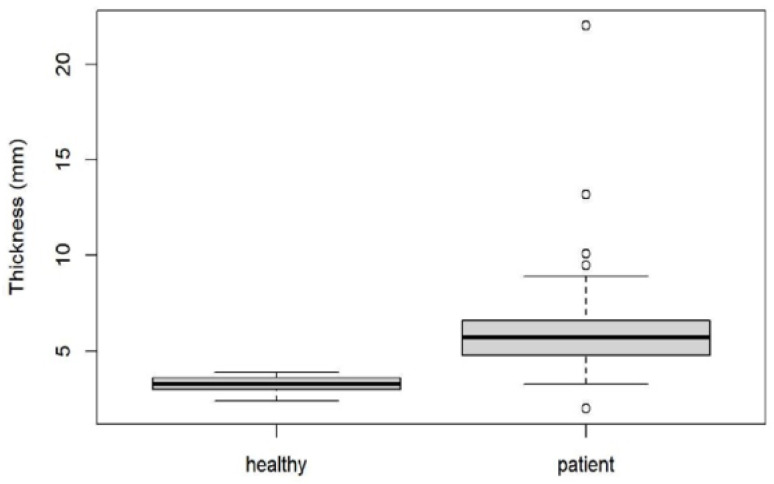
Box blot diagram showing plantar fascial thickness in healthy volunteers compared to patients with plantar fasciitis.

**Figure 5:**
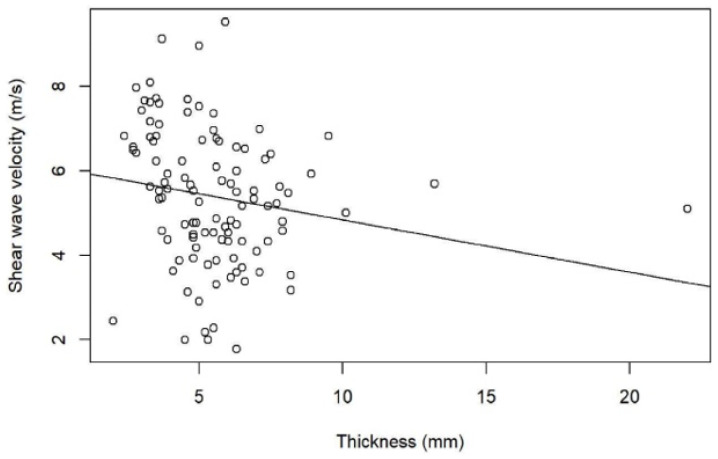
SWV and thickness for Patients with plantar fasciitis and Healthy volunteers, showing no correlation.

We found a mean thickness in healthy plantar fasciae of 3.28 mm (SD: 0.41, range: 2.4–3.9) compared to 6.07 mm (SD: 2.37, range: 2.0–22.0) in plantar fasciitis (*p* < 0.001) (Figure 1 and Figure 4). There was no correlation between SWV and plantar fascial thickness (r² = 0.02, *p* = 0.06) or between stiffness and plantar fascial thickness (r² = 0.02, *p* = 0.06) (Figure 5).

For thickness the obtained cut-off upper normal limit was 4.0 mm (AUC: 0.97, 95% CI: 0.94–0.99) with a specificity of 100% (95% CI: 100–100%) and a sensitivity of 90.8% (95% CI: 83.91–96.55%). No concomitant plantar fascia tears were found by B- mode US.

CDUS showed no correlation to stiffness and SWV (r² < 0.007, *p* = 0.4) and there was no significant difference for stiffness or SWV values between e.g., CDUS grade 0 and grade 3 (*p* = 0.2).

**Figure 6:**
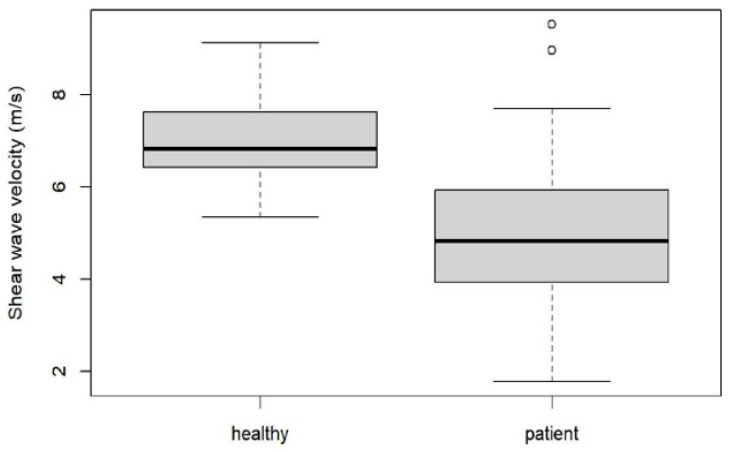
Box blot diagram showing SWV in healthy volunteers compared to patients.

**Figure 7:**
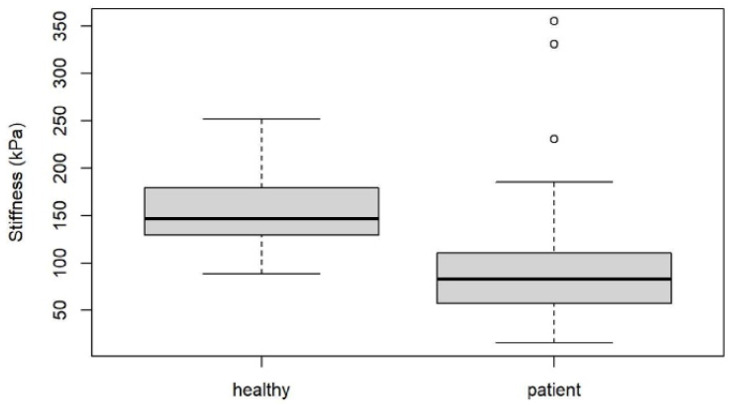
Box blot diagram showing Stiffness in healthy volunteers compared to patients.

Mean SWE and stiffness values for healthy plantar fascia and plantar fasciitis are shown in Figure 6 and Figure 7, and Figure 2 and Figure 3 with an intra class correlation of 0.43–0.64 for SWE and 0.42–0.63 for SWV.

There was a statistically significant difference in SWV and plantar fascial stiffness between healthy subjects and patients with plantar fasciitis (*p* < 0.001).

For SWV, ROC analysis resulted in a cut-off value of 6.16 m/s (AUC: 0.87, 95% CI: 0.80–0.94), giving a specificity of 80.95% (95% CI: 61.9–95.24%) and a sensitivity of 79.31% (95% CI: 70.11–87.36%). For stiffness a cut-off value of 125.57 kPa (AUC: 0.85, 95% CI: 0.77–0.92) was found with a specificity of 80.95% (95% CI: 61.9–95.24%) and a sensitivity of 80.46% (95% CI: 72.39–88.51%)

## 4. Discussion

Our results by using SWE confirmed softening of the plantar fascia in the patient group when compared to healthy volunteers, which already has been demonstrated by using SEL in recent studies [22,23,25].

Sconfienza et al. [23] demonstrated that the use of real-time SEL increases the diagnostic performance of B-mode US and may also be helpful in some cases in which the results of B-mode US are inconclusive. Their study confirmed that B-mode US can demonstrate specific signs of plantar fasciitis. Of those, fascia thickening and hypoechoic echotexture were more typical in plantar fasciitis than blurring of the fascial borders, which seems to be a less reproducible finding.

Our study confirms that B-mode US is still the most reliable diagnostic tool for plantar fasciitis diagnosis. Our thickness measurement results are in line with the literature, where a plantar fascial thickness greater than 4 mm has been postulated to be consistent with plantar fasciitis. A lack of standardization of the measurement process for plantar fascia thickness might limit the measurements. In particular, there are no universal guidelines existing on the positioning of the metatarsophalangeal (MTP) joints during the procedure and the literature also has inconsistent protocols [28]. We used the relaxed feet positioning, which is comfortable for the patient and examiner on the one site and minimizes errors through muscle tension on the other site [24].

Wu et al. showed that SEL was helpful in the diagnosis of plantar fasciitis in patients presenting with normal B-mode US thickness measurements [22,25]. In our study we found no correlation between SWE and plantar fascial thickness (r² = 0.02, *p* = 0.06) or between stiffness and plantar fascial thickness (r² = 0.02, *p* = 0.06). This is in line with the results of Wu et al.

CDUS has already been found with poor sensitivity in plantar fasciitis patients, which is in line with our results [29].

Putz et al. 2017 found that contrast enhanced US (CEUS) improved detection of hyperemia in 75% of patients and advocated CEUS as well as SWE as new diagnostic tools in the assessment of plantar fasciitis proving helpful for quantitative parameters and monitoring therapy [30].

Gatz et al. 2019 showed that SWE can improve the diagnostic accuracy in patients with plantar fasciitis compared to B-mode US. He found in healthies with a thickness of 3 mm and in patients, where the plantar fascia was thicker than 4.2 mm a cut off of 51.5 kPa and 4.14 m/s. He also showed lower values for plantar fasciitis of 31.9 kPa and 3.26 m/s and statistical significant higher values in asymptomatic of 93.3 kPa and 5.58 m/s with a sensitivity of 85% and specificity of 83% for SWE and a B-mode US sensitivity of 61% specificity of 95% [18].

One limitation of the study by Gatz et al. is that healthy volunteers were statistically significant different in age compared to the 39 fasciitis patients, therefore an age dependency might explain the difference to our results, which has already been demonstrated by Wu et al. showing that plantar fascia softens with age and in subjects with fasciitis [25]. Gatz also stated, that he worked with a relatively small sample group. Therefore our study adds valuable data. Our study had several limitations:

SWE values obtained on an Aixplorer system may not be equivalent to the SWE values obtained on other systems.

A small area analyzed on the color histogram does not represent the entire insertion of the plantar fascia. This would have been covered by axial transducer placement. 

However, the thickest part of the plantar fascia was evaluated by SWE as detected by B-mode in longitudinal plane which is usually the proximal central band. Furthermore, because of the not parallel course of the fascia in that area, we adjusted the position and the tilting angle of the transducer to avoid anisotropy on B-mode sonogram and to obtain SWE measurements. Furthermore, no histologic testing was performed in this study. Further histopathologic and biomechanical examinations is required confirm our results. Our study has demonstrated SWE to be a reliable, in vivo noninvasive technique for examining the stiffness of the plantar fascia.

We did not have any other imaging as a reference. No measurements of the contralateral plantar fascia as an intern standard have been performed. We did not calculate a ratio. Finally, the healthy volunteers were referred from Hospital staff without any symptoms at the feet, what might have been a bias in the sampling.

## 5. Conclusions

SWE proved to be a valuable tool in the detection of plantar fasciitis and may be helpful in addition to B-mode US thickness measurement in unclear cases.
